# CT, MRI and DWI Features of a Solid Organizing Hepatic Abscess

**DOI:** 10.1155/2014/930569

**Published:** 2014-08-13

**Authors:** Sergio Savastano, Giampiero Pellizzer, Lorenzo Di Grazia, Dario Giacomini, Mario Beghetto

**Affiliations:** ^1^Department of Radiology, San Bortolo Hospital, Viale F. Rodolfi 37, 36100 Vicenza, Italy; ^2^Department of Infectious Diseases, San Bortolo Hospital, Viale F. Rodolfi 37, 36100 Vicenza, Italy

## Abstract

Solid organizing hepatic abscess is a rare form of focal infection, which needs differentiation from benign and malignant solid masses. We report a case of a 30-year-old man with a solid organizing hepatic abscess, diagnosed by imaging and *ex juvantibus* criteria. CT and MRI findings are presented and role of DWI is outlined. Noninvasive diagnosis of a solid organizing hepatic abscess is possible in the appropriate clinical setting; percutaneous or surgical biopsy may be indicated in equivocal cases.

## 1. Introduction

Cross-sectional imaging is a reliable tool for diagnosing hepatic abscesses in the appropriate clinical setting, but because of nonspecific radiological features percutaneous biopsy may be required for a definitive diagnosis in patients with equivocal clinical symptoms [1, 2] especially in cases of solid abscesses which may mimic a solid tumor [3–7].

Among solid infected lesions of the liver, usually due to pyogenic pathogens,* Brucella* or parasites [4–7], organizing hepatic abscesses show peculiar features on computed tomography (CT) and magnetic resonance imaging (MRI) scans, which may suggest the correct diagnosis [8].

We here present a case of an organizing hepatic abscess diagnosed on the basis of cross-sectional imaging and* ex juvantibus* criteria; features and role of diffusion weighted imaging (DWI) are also described.

## 2. Case Report

A 30-year-old man with an unremarkable past clinical history was hospitalized for intermittent fever lasting for three weeks and a hypoechoic mass in the hepatic segment IV detected by an abdominal ultrasonography performed in an ambulatory diagnostic clinic. He was treated at home with ciprofloxacin and amoxicillin-clavulanate without clinical benefit. Laboratory tests at admission revealed neutrophil granulocytosis (WBC 16.3 × 10^9^/L with 78% neutrophils), increased C-reactive protein level (19.9 mg/dL; upper normal limit of 0.50 mg/dL), and mild increase of transaminases (P-AST 91 U/L, P-ALT 171 U/L; upper normal levels 37 U/L and 53 U/L, resp.). Urinalysis was normal. Sera IgA, IgG, and IgM and tumor markers were within normal limits. Repeated blood culture was negative as well as serological test for viruses (HIV, HBV, and HCV), parasites (*Entamoeba histolytica*), and bacteria (*Bartonella*, Q fever, and* Mycoplasma pneumoniae*). Autoantibody tests (antinuclear antibodies and antineutrophil cytoplasmic antibody) for systemic autoimmune disease were negative. After admission he was treated with piperacillin-tazobactam (4.5 gr t.i.d, i.v.) for 2 weeks and then p.o. with levofloxacin and amoxicillin-clavulanate for further 2 weeks; no steroids were administered. Fever definitely resolved on the third day after admission.

**Figure 1:**
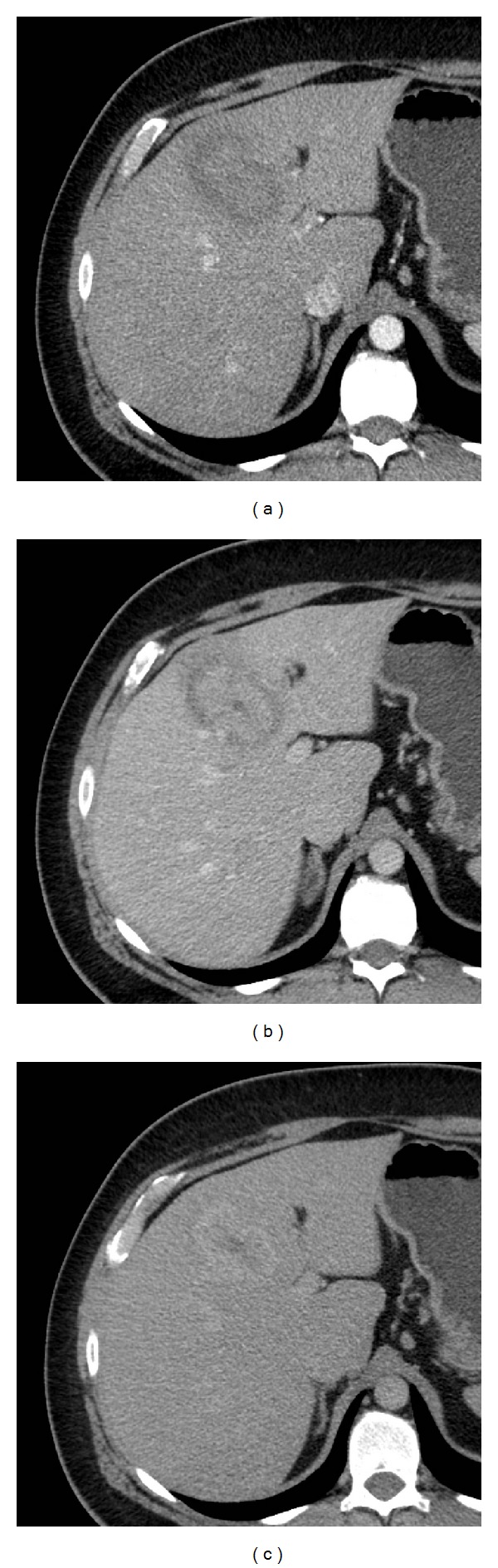
Abdominal contrast-enhanced CT. (a) and (b) Scans on arterial and portal phases show a target-like lesion in the segment IV of the liver. The lesion and the normal hepatic parenchyma enhance likewise; a hypoattenuating rim and a tiny hypoattenuating core are also evident. (c) The rim strongly enhances on late venous phase scans.

A CT examination of the abdomen was performed with a 64-multislice equipment (VCT pro, GE, Milwaukee, USA) before and after intravenous injection of 120 mL of iopromide 370 mg/mL at the rate of 4 mL/sec (Ultravist 370 Bayer, Berlin, Germany). Nonenhanced CT scans showed a hypoattenuating lesion measuring 6.5 × 4 cm in the hepatic segment IV. The lesion exhibited a target appearance on dynamic multiphasic CT scans, the main central component showing an enhancement similar to normal hepatic parenchyma. This component was circumscribed by a rim which was conversely hypoattenuating on arterial and venous phases and hyperattenuating on late venous phase. The lesion contained a tiny colliquative core (Figure 1).

One week later, during i.v. antibiotic therapy, a MRI of the liver was performed with a 1.5T magnet (Avanto, Siemens Healthcare, Erlangen, Germany) and a 16-channel phased-array body surface coil using the following sequences protocol: axial in-phase and out-of-phase GE T1, coronal and axial HASTE T2-weighted imaging, axial fat suppressed HASTE T2-weighted sequence, DWI at three different* b*-values (50, 400, and 800 s/mm^2^), and nonenhanced and contrast-enhanced dynamic spoiled 3D GE sequence before and after i.v. injection of gadobenate dimeglumine (0.1 mL/body weight kg, Multihance; Bracco, Milano, Italy); the biliary phase was not acquired.

**Figure 2:**
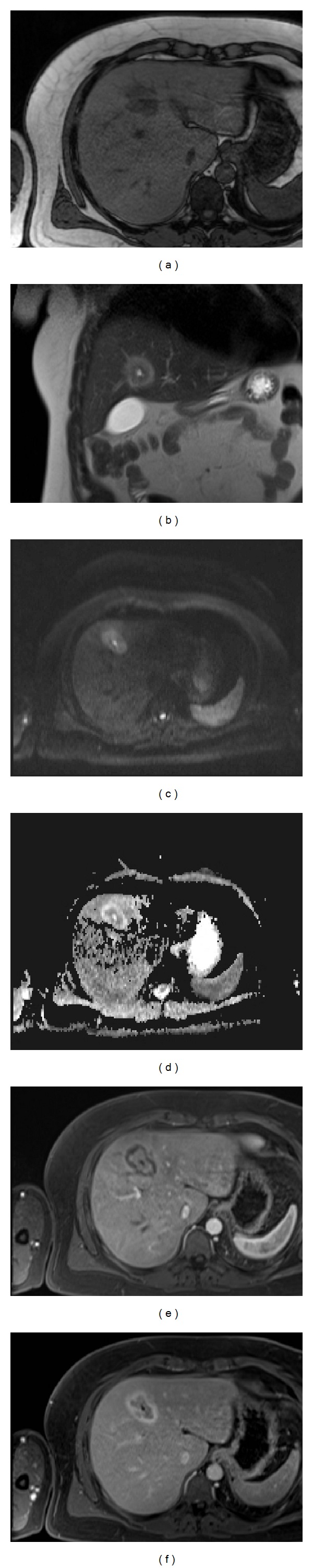
MRI of the liver. (a) The nodular mass is hypointense on axial GE T1-weighted out-of-phase MRI. (b) The lesion exhibits a target-like appearance on coronal T2-weighted HASTE MRI, the rim being higher in signal intensity than the central component. The tine colliquative core is clearly visible. (c) The central component is hyperintense on DWI_*b*400_, while the rim is not appreciable; the colliquative nucleus and the cerebrospinal fluid are hyperintense because of T2-shine-through effect. (d) ADC map shows no water diffusion restriction of the rim and the core. (e) and (f) Contrast-enhanced pattern of the nodule on dynamic MRI (arterial and late venous phases) is similar to the pattern of dynamic CT.

MRI documented a decrease in the size of the lesion, which now measured 5 × 3 cm. The lesion was hypointense on T1-weighted MRI and hyperintense on T2-weighted MRI; in the latter sequence, the lesion showed a target-like appearance, the rim being higher in signal than the central component; moreover, the core was highly hyperintense (Figures 2(a) and 2(b)). DWI demonstrated a partial water diffusion restriction of the main component; the mean ADC values of the rim, the central component, the colliquative core, and the normal liver were 1920 × 10^−3^ mm^2^/s, 1080 × 10^−3^ mm^2^/s, 1930 × 10^−3^ mm^2^/s, and 870 × 10^−3^ mm^2^/s, respectively (Figures 2(c) and 2(d)). Lesion enhancement on dynamic MRI resembled contrast-enhanced CT appearance (Figures 2(e) and 2(f)).

A percutaneous biopsy was scheduled but not performed because the patient refused to consent. Abdominal ultrasonography documented further decrease in size of the lesion 4 days after MRI examination (3 cm in larger diameter).

Patient recovered completely during the hospitalization and all laboratory tests were normal at the time of discharge. No liver abnormalities were appreciable on 2-month MRI follow-up. Final diagnosis of an organizing hepatic abscess was made on the basis of diagnostic imaging and* ex juvantibus* criteria.

## 3. Discussion

Organizing solid abscesses of the liver are lesions with unknown incidence and gender predilection, pathologically characterized by a prominent chronic inflammatory reaction circumscribed by a fibrous rim and centered on a tiny necrotic suppurative core, not always recognizable on diagnostic imaging; biliary stone disease and recurrent pyogenic cholangitis are considered predisposing factors [8]. Histological features and absence of bacteria microscopically identifiable suggest an atypical slow-healing process in patients partially responsive to antibiotic therapy [8]. Absence of a large colliquative cavity precludes these lesions from treatment by percutaneous drainage [4].

Kim et al. described CT and MRI findings of solid organizing hepatic abscesses, which characteristically exhibit a target appearance, more conspicuous on T2-weighted MRI and dynamic imaging [8]. The prominent inflammatory component and the peripheral rim, respectively, isointense and hyperintense with respect to the liver on T2-weighted MRI, show contrary features on dynamic imaging. The central area enhances similarly to the normal liver on arterial and portal phases of dynamic CT scans and MRI and becomes hypoattenuating/hypointense on delayed scans, whereas the fibrous rim enhances in the late phases only [8]. In our case, the large inflammatory area showed no significant water diffusion restriction on DWI with respect to the liver; also high ADC values of the rim suggested the benign nature of the lesion [9].

Pyogenic hepatic abscesses may be solid on diagnostic imaging [6, 10]. Alsaif et al. reported an incidence of predominantly solid abscesses on monophasic contrast-enhanced CT scans in 57% of cases of monomicrobial* Klebsiella pneumoniae* infection versus 36% of cases of polymicrobial infections, with a 90% positive pus culture rate for both groups; these abscesses are however “predominantly” solid and are not circumscribed by a rim [6]. These findings suggest that such abscesses are discrete entities compared to those described by Kim et al. [8], in which an idiosyncratic inflammatory reaction perhaps plays a main role.

Hepatic abscesses may present as solid or target-like lesion in patients with chronic granulomatous disease, an inherited immunodeficiency of childhood characterized by primary phagocyte defect [4]. These abscesses are recurrent and often multiple and present no or tiny liquefaction nucleus in early stages; the rim of these lesions strongly enhances on postcontrast imaging but the main solid component usually enhances less than the surrounding hepatic parenchyma [4, 11, 12].

Organizing solid hepatic abscesses should be also differentiated from solid masses. Hepatic inflammatory pseudotumors are characterized by a large spectrum of CT and MRI findings, sometimes with a multilayer feature [13, 14]. They are usually hypointense and hyperintense on T1-weighted and T2-weighted MRI, respectively, and do not enhance on arterial phase of dynamic imaging but only on delayed scans [8, 13].

Primary and metastatic hepatic tumors should also be included in differential diagnosis. Neoplasms have high signal intensity on T2-weighted MRI, high* b-*value DWI, and low ADC values. Hypervascular primary and metastatic tumors enhance more than the normal hepatic parenchyma on arterial images whereas cholangiocarcinoma and hypovascular metastases enhance during the late phases [8]. Moreover, tumorous rim, when present, shows ADC values lower than abscess rim [9].

In conclusion, the diagnosis of solid organizing hepatic abscess may be suggested by specific CT and MRI findings in the appropriate clinical setting. Nevertheless, percutaneous biopsy is mandatory for a definitive diagnosis in equivocal cases.

## References

[1] Mathieu D, Vasile N, Fagniez PL, Segui S, Grably D, Lardé D (1985). Dynamic CT features of hepatic abscesses. *Radiology*.

[2] Mortelé KJ, Segatto E, Ros PR (2004). The infected liver: radiologic-pathologic correlation. *Radiographics*.

[3] Gabata T, Kadoya M, Matsui O (2001). Dynamic CT of hepatic abscesses: significance of transient segmental enhancement. *American Journal of Roentgenology*.

[4] Garcia-Eulate R, Hussain N, Heller T (2006). CT and MRI of hepatic abscess in patients with chronic granulomatous disease. *American Journal of Roentgenology*.

[5] Chourmouzi D, Boulogianni G, Kalomenopoulou M, Kanellos I, Drevelegas A (2009). Brucella liver abscess; imaging approach, differential diagnosis, and therapeutic management: a case report. *Cases Journal*.

[6] Alsaif HS, Venkatesh SK, Chan DSG, Archuleta S (2011). CT appearance of pyogenic liver abscesses caused by Klebsiella pneumoniae. *Radiology*.

[7] Mukund A, Arora A, Patidar Y (2013). Eosinophilic abscesses: a new facet of hepatic visceral larva migrans. *Abdominal Imaging*.

[8] Kim YK, Kim CS, Lee JM, Ko SW, Moon WS, Yu HC (2006). Solid organizing hepatic abscesses mimic hepatic tumor: multiphasic computed tomography and magnetic resonance imaging findings with histopathologic correlation. *Journal of Computer Assisted Tomography*.

[9] Park HJ, Kim SH, Jang KM, Lee SJ, Park MJ, Choi D (2013). Differentiating hepatic abscess from malignant mimickers: value of diffusion-weighted imaging with an emphasis on the periphery of the lesion. *Journal of Magnetic Resonance Imaging*.

[10] Tan CB, Shah M, Rajan D (2012). A solid organising cryptogenic liver abscess and its association with a colonic tubullovillous adenoma. *BMJ Case Reports*.

[11] Lublin M, Bartlett DL, Danforth DN (2002). Hepatic abscess in patients with chronic granulomatous disease. *Annals of Surgery*.

[12] Leiding JW, Freeman AF, Marciano BE (2012). Corticosteroid therapy for liver abscess in chronic granulomatous disease. *Clinical Infectious Diseases*.

[13] Anderson SW, Kruskal JB, Kane RA (2009). Benign hepatic tumors and iatrogenic pseudotumors. *Radiographics*.

[14] Rosa B, Moutinho-Ribeiro P, Pereira JM (2012). Ghost tumor: an inflammatory pseudotumor of the liver. *Gastroenterology and Hepatology*.

